# Paradox of diversity in the collective brain

**DOI:** 10.1098/rstb.2020.0316

**Published:** 2022-01-31

**Authors:** Robin Schimmelpfennig, Layla Razek, Eric Schnell, Michael Muthukrishna

**Affiliations:** ^1^ Department of Organizational Behavior, University of Lausanne (UNIL), Chavannes-près-Renens, Lausanne 1015, Switzerland; ^2^ Department of Biology, McGill University, Dr Penfield Avenue, Montreal, Canada H3A 1B1; ^3^ Department of Psychological and Behavioural Science, London School of Economics and Political Science (LSE), Houghton Street, London WC2A 2AE, UK; ^4^ Canadian Institute for Advanced Research, Toronto, Ontario, Canada M5G 1M1

**Keywords:** cultural evolution, diversity, collective intelligence, collective brain, innovation, evolvability

## Abstract

Human societies are collective brains. People within every society have cultural brains—brains that have evolved to selectively seek out adaptive knowledge and socially transmit solutions. Innovations emerge at a population level through the transmission of serendipitous mistakes, incremental improvements and novel recombinations. The rate of innovation through these mechanisms is a function of (1) a society's size and interconnectedness (sociality), which affects the number of models available for learning; (2) fidelity of information transmission, which affects how much information is lost during social learning; and (3) cultural trait diversity, which affects the range of possible solutions available for recombination. In general, and perhaps surprisingly, all three levers can increase and harm innovation by creating challenges around coordination, conformity and communication. Here, we focus on the ‘paradox of diversity’—that cultural trait diversity offers the largest potential for empowering innovation, but also poses difficult challenges at both an organizational and societal level. We introduce ‘cultural evolvability’ as a framework for tackling these challenges, with implications for entrepreneurship, polarization and a nuanced understanding of the effects of diversity. This framework can guide researchers and practitioners in how to reap the benefits of diversity by reducing costs.

This article is part of a discussion meeting issue ‘The emergence of collective knowledge and cumulative culture in animals, humans and machines’.

## 1. Introduction

Innovation is often assumed to be the work of a talented few—the giants upon whose shoulders we stand. This assumption, however, is inconsistent with theoretical and empirical research in cultural evolution [1,2] which instead suggests that innovation is more accurately described as an emergent property of our species' cultural learning psychology, applied within our societies and social networks. Human societies and social networks form ‘collective brains’ such that innovations emerge at a population level requiring a specific innovator no more than our thoughts require a specific neuron. Indeed, not only is the world too complicated for even the smartest among us to recreate it is also more complicated than our psychology allows us to believe.

People are unaware that at best they possess a partial causal model of most of the world they interact with, what has been referred to as the ‘illusion of explanatory depth’ [3–5]. But as recent experiments reveal, a lack of causal understanding does not prevent solutions from accumulating through selective social learning [6]. Partial causal models can drive incremental improvement, but large innovative leaps are rarely a product of causal cogitation, instead, they are typically driven by serendipity and recombination of existing ideas [1].

Incremental improvement, serendipity and recombination are influenced by three key levers: sociality, transmission fidelity and cultural trait diversity. In the next section, we discuss the challenges that must be resolved for each lever to increase innovation. This paper focuses on the effects and challenges of diversity, in particular cultural trait diversity. Diversity is fuel for recombination. Recombination has far more potential to drive innovation than incremental improvement or luck. But to reap the benefits of cultural trait diversity, researchers and practitioners need to better understand how diversity affects innovation both in terms of potential benefits and potential costs.

We discuss the interdisciplinary theoretical and empirical literature on the relationship between cultural trait diversity and innovation in terms of the ‘paradox of diversity’—that diversity is both fuel for recombination and a challenge to communication and coordination. We present a formal model that captures this trade-off in the collective brain. We then introduce the concept of ‘cultural evolvability’ as a framework for understanding and resolving the paradox. We illustrate the insights using the evolution of overconfidence and its implications for entrepreneurship. These topics are of interest to both basic and applied scientists working on cultural evolution and innovation. In the final section, we focus on the policy implications of this approach to reaping the benefits of diversity while minimizing the costs.

## 2. Trade-offs in the collective brain

**Figure 1.**
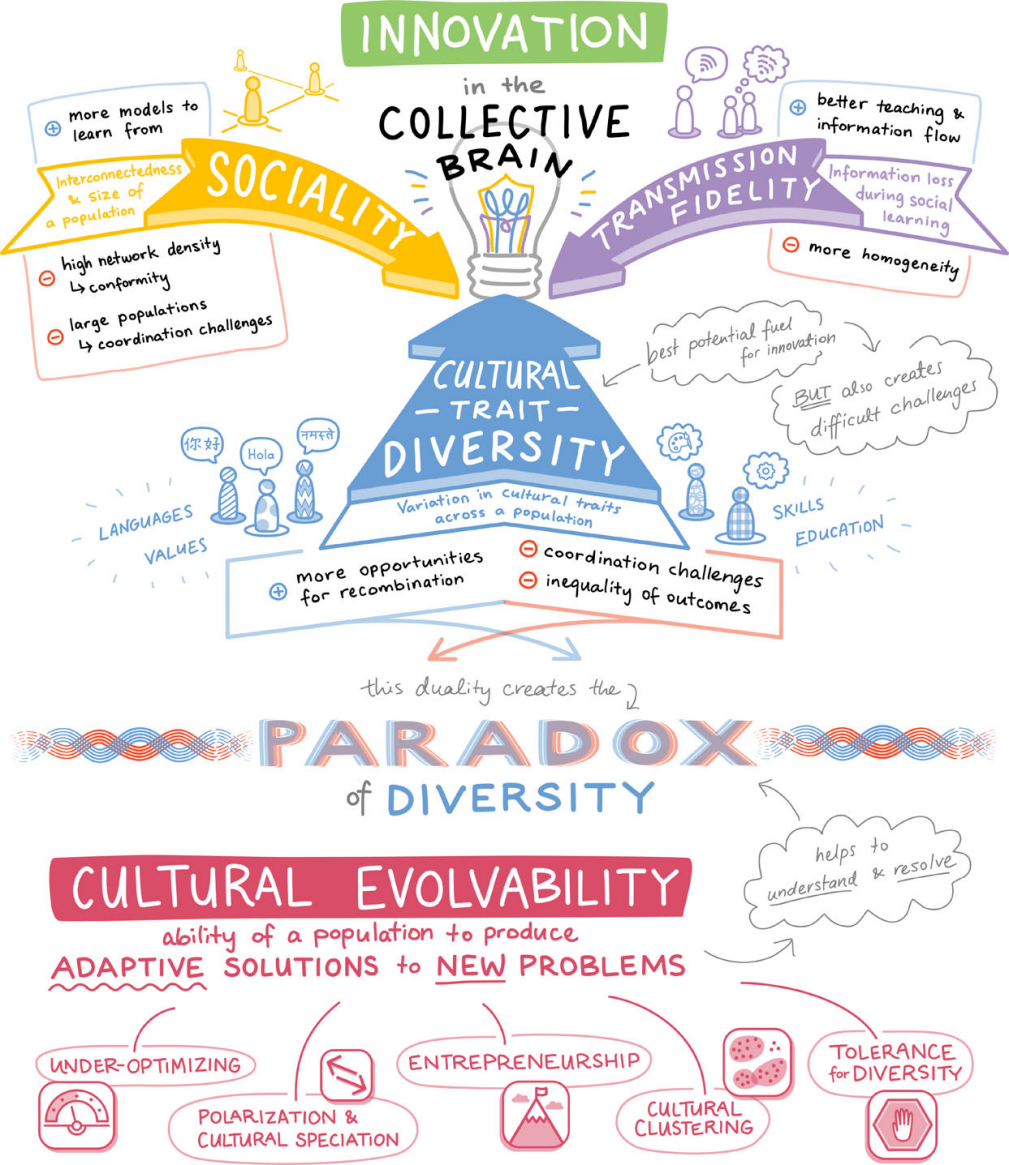
Innovation in the collective brain is influenced by three levers: sociality, transmission fidelity and cultural trait diversity. All three levers can increase and harm innovation. Cultural trait diversity offers the most potential, but also a difficult challenge. This duality of cultural trait diversity creates the paradox of diversity. We introduce cultural evolvability as a means to better understand and resolve the paradox of diversity. (Online version in colour.)

Each lever of the collective brain—sociality, transmission fidelity and cultural trait diversity—presents trade-offs and challenges to innovation (summarized in figure 1). Here, we discuss these challenges, how they are resolved, and why cultural trait diversity offers the most potential, but also a difficult challenge.

### (a) Sociality

Sociality describes the size and interconnectedness of a society—larger, more interconnected societies offer more people from whom to learn and have more ideas that can more easily flow through denser social networks to meet and recombine. Early theoretical models [7,8] predicted a positive relationship between sociality and cultural complexity. This predicted pattern was supported by correlational [9] and later experimental evidence [10–12]. But this straightforward positive relationship has some caveats.

Mesoudi [13], for example, models variable learning costs in cultural traits, predicting an asymptotic relationship between sociality and cultural complexity. Some traits are more difficult to learn, decreasing transmission fidelity as cultural complexity increases. Mesoudi offers the example of mathematics and science. Unless they get a PhD, twenty-first-century students typically do not learn any mathematics developed after 1900; scientific training takes longer, and major contributions are made at an older age.

The relationship predicted by Henrich [7] also assumes sufficiently difficult skills that must be socially (rather than individually) learned. Sociality would not predict improved performance in sufficiently simple tasks (for discussion, see [12]).

Increases in population size can also create coordination challenges and increases in interconnectivity can reduce diversity through conformity. Indeed, more recent theoretical and empirical research suggests a non-monotonic relationship between sociality and cultural complexity [14–19]. Too small a population means too few models to learn from, but too large a population creates a coordination challenge reducing effective sociality.^1^ Too little interconnectedness also means too few models to learn from, but too high interconnectedness poses a coordination challenge and risks reducing diversity through conformity. The resolution to this apparent contradiction is twofold.

First, as societies grow they evolve cooperative substructures such as departments, firms and regional governments that reduce the coordination challenges relative to a flat structure [20]. Indeed, given that smaller cooperative groups can undermine larger cooperative groups [20–22], resolving these challenges may be a requirement for large cooperative populations to thrive. We see a micro version of this process in organizations. As organizations grow, so too do the challenges of communication and coordination. Organizations learn from one another, modifying and implementing a variety of policies and organizational structures from flat to hierarchical to matrixed in attempts to resolve these challenges [23].

Second, sociality is a function of both group size and interconnectedness [7,24]. Muthukrishna & Henrich [1] argue that there exists an optimal interconnectedness. Large populations (e.g. cities and countries) have low network density and low interconnectedness and thus benefit from increases in connectivity. By contrast, small populations (e.g. corporate teams, groups in psychology experiments) may be easily overconnected, increasing coordination challenges and reducing diversity through conformity. These challenges of communication, coordination and conformity overlap with both the challenges of transmission fidelity and cultural trait diversity.

### (b) Transmission fidelity

Transmission fidelity refers to the degree of information preservation during social learning and is therefore increased by better means of communication. Early genetically evolved and culture–gene coevolved improvements to transmission fidelity may have included joint attention and shared intentionality [25,26], theory of mind [27], social tolerance and prosociality [20,28], and sophisticated language [29–32]. Later culturally evolved improvements include information compression through heuristics and biases, easier learning through simplified steps, the discovery and spread of fundamental principles that support triangulation, and teaching. Muthukrishna *et al*. [33] argue that improved transmission fidelity is under selection in support of keeping up with an ever-growing body of cumulative cultural information. Observed differences in teaching practices over history and between societies support this argument [34]. Explicit and effortful teaching covaries with cultural complexity.

In many hunter–gatherer societies, teaching occurs by allowing children to observe, perhaps slowed-down, actions [34–38]. More explicit and effortful instruction is observed among many pastoralist societies and compulsory formal education emerged as a response to the Industrial Revolution. Migliano & Vinicius [19] make a complementary argument that teaching in small-scale societies evolves with growing tendencies toward pair bonding and shared reproductive interests, arguing that cooperation with unrelated individuals can decrease the cost–benefit ratio of learning more complex technologies and social norms. Cultural evolution continues to increase transmission fidelity in our increasingly culturally complex modern world, through technologies such as the printing press, radio, television, Internet, video conferencing and social media.

We therefore expect a mechanistic positive relationship between transmission fidelity and innovation. That is, societies require improvements in transmission fidelity to support greater cultural complexity. However, high transmission fidelity can also reduce cognitive and cultural diversity and increase ‘Global WEIRDing’ (WEIRD: Western, educated, industrialized, rich and democratic; [39–43]). There is also a limit to improvements to cultural complexity via improving transmission fidelity alone. For example, one solution is more time to learn such as through a cultural extension of the juvenile period. But this extension of the time required for sufficient education to survive and thrive in an industrialized society requires additional support at an older age, increases the time to peak productivity, and delays the age of reproduction [1]. These limits mean that improvements in transmission fidelity alone are insufficient to support continuing increases in innovation. Another solution is to simply divide the information (and labour) among different people—specialization—creating cultural trait diversity. Cultural trait diversity can continue to support increases in innovation as long as sociality is sufficient to ensure enough specialists in every domain.

### (c) Cultural trait diversity

Diversity comes in different types measured in different ways [44]. We focus on cultural trait diversity—differences in beliefs, behaviors, assumptions, values, technologies and other transmissible traits. This could include languages, processing techniques and technical skills, but also broader traits such as family structure and occupation. In the public discourse, diversity often refers to ancestry or physical characteristics. These may correlate with cultural trait diversity, though the correlation may weaken over generations through acculturation [45]. Here ‘diversity’ refers to cultural trait diversity unless otherwise specified. Cultural trait diversity can be distributed in different ways.

Diversity between populations culturally evolves as populations adapt to local differences, influencing future generations through historical path dependence created by past conditions or founder populations [46–48]. Diversity within populations evolves as information and labour are divided [49,50]; a way to handle an ever-growing corpus of cumulative culture (see the model in the electronic supplementary material). Within-population diversity includes disciplinary differences, such as the sciences and humanities, industry specializations [51], guilds and firms [41]. Diversity can also be structured as ‘cultural clusters’ by ethnicity, class, wealth, occupation, political alignment, religion or incidental geographical layout [52]. Cultural clusters may intersect, such as in ethnic occupation specialization [53]. Finally, cultural trait diversity may also exist within certain individuals—multicultural individuals, ‘third culture kids’, interdisciplinary researchers and so on [54–58].

Cultural trait diversity is therefore both the product of cultural evolution and fuel for the engine of further innovation. However, like sociality and transmission fidelity, it also comes with a cost [59]. Without a common understanding and common goals, the flow of ideas in social networks is stymied, preventing recombination and reducing innovation. As obvious examples, consider the challenge of communication without a common language or of collaborations between scientists and humanities scholars or even between scientists from different disciplines.

But in contrast to sociality and transmission fidelity, which have fundamental limits, cultural trait diversity has a much greater scope as fuel for continuing human innovation. Recombination through diversity offers almost unlimited innovation potential but diversity can also make it difficult to communicate and coordinate: the paradox of diversity.

### (d) Modelling the paradox of diversity

To better understand the paradox of diversity, we present a formal model of the trade-off that arises from the division of labour. In this model, a group of *N* individuals is faced with learning *M* domains of knowledge with a limited brain and cognitive capacity *b*. At one extreme, individuals become experts in a single domain. This allows them to achieve greater skill in this single speciality (*b*), but makes it difficult for two individuals to coordinate, having no overlap in knowledge. At the other extreme, everyone learns all domains, but given their limited *b* brain, they learn very little about every domain (*b/M*). This removes the coordination problem but leaves the group with a low level of knowledge in each domain. Simply put, the division of labour involves a trade-off between coordination efficiency and increasing skill levels. More ideas and ways of thinking on the one hand and difficulties in coordinating, communicating, and agreeing on goals on the other.

**Figure 2.**
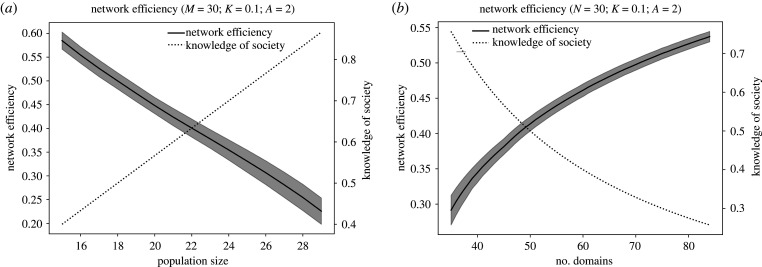
We simulate the trade-off of knowledge of society (skill levels) and network efficiency (coordination). Each player learns a certain number of domains and makes a connection to other members with an overlap in skills. Coordination in the society is then measured as the ease of traversing this network. The solid curve represents network efficiency and the dotted curve represents the knowledge of the society (see electronic supplementary material for details on how these are calculated). As population size increases (*a*) network efficiency decreases and the knowledge of the society increases. As the number of domains being learnt increases (*b*) network efficiency increases and the knowledge of the society decreases. When a change occurs in population size or the number of domains, the other variable can resolve this trade-off at a new optimum. For instance, if population size increases, a society can learn more skills to improve coordination.

Figure 2 shows the results of this modelled trade-off (details in the electronic supplementary material). As population size increases (figure 2*a*) the coordination problem is exacerbated and the skill level increases. As the number of domains being learnt increases (figure 2*b*) coordination improves and the skill level decreases. When a change occurs to one of these variables, the other variable must adapt to resolve this trade-off. For instance, when population size increases, people can specialize in fewer domains with the same level of coordination, but increased innovation. Thus, greater cultural trait diversity requires greater sociality. For example, in a small town, there may be a single general physician who needs to know many domains of medicine. But in New York, a doctor may specialize in a small part of the renal system and get very good at treating that one part, because other specialists cover other domains. New York populated entirely by nephrologists would not survive long.

Increasing coordination will allow for greater specialization and greater cultural trait diversity, supporting innovation. An important question of scientific and practical importance is thus how to reap the recombinatorial benefits of diversity in the collective brain, without paying possible coordination costs.

We turn to the evolutionary biological literature on evolvability for how to understand this paradox and wield diversity's double-edged sword. We can apply this literature to cultural evolution and the collective brain to develop the concept of ‘cultural evolvability’.

## 3. Cultural evolvability applied to the paradox of diversity

Evolvability refers to the ability of a biological system to produce heritable, adaptive solutions [60,61]; we focus on the evolvability of a population rather than specific traits [62–64]. A population may be highly adapted to the local environment, but may or may not have the evolvability to adapt to changes in the environment. For example, consider Darwin's finches with beak shapes and sizes that are optimized for the present nut shapes and sizes. For the range of nut shapes and sizes, there is an optimal range of beak shapes and sizes. But if the distribution of nut shapes changes, then the ability of the finches to evolve beak shape or size to compensate will depend on their evolvability. Variation or diversity, and the forces that create and stabilize that diversity are key factors that create evolvability.

Cultural evolvability is a balance between diversity and selection, exploring and exploiting, sampling and specializing, convergent and divergent thinking, stability and change, efficiency and flexibility. If a system features an abundance of diversity, some of the traits are necessarily less adaptive than others. But without that range of traits when the environment shifts, there would be an inability to adapt. Thus, ensuring evolvability necessarily means accepting some amount of inequality and a population-level payoff less than the current potential maximum. Within population genetics, many open questions remain on how evolvability itself evolves [60,61,65–68]. That is, not how organisms find the most adaptive traits, but how populations or biological systems support or generate the diversity necessary to adapt when circumstances change.

Several papers implicitly tackle the cultural evolvability trade-off between diversity and selection in cultural innovation and adaptation [1,7,63,69–71]. These are analogous to the explore–exploit or sampling–specializing trade-off in development over the lifespan [72,73] and the search for global solutions and avoidance of saddle points within machine learning [74].^2^

Cultural evolvability refers to the ability of a society to culturally evolve to changed circumstances. It offers a framework for understanding and resolving the paradox of diversity. For example, cultural evolvability can help us to understand optimal levels of diversity or how populations can reap the benefits of cultural trait diversity by reducing diversities' coordination cost. What are these costs and benefits and how can cultural evolvability help us understand them?

Here, we review the theoretical and empirical literature on the effects of diversity in different settings through the lens of cultural evolvability. But diversity comes in many forms and interpreting the diverse literature on diversity poses several challenges:

1. Definitions of diversity differ between academic fields, and between academia and the general public.
2. Even where definitions are similar, the measurement may differ.
3. Results are sometimes causal and sometimes correlational.
4. Several causal pathways may exist in parallel. For example, education creates cultural differences (e.g. low and high education), but independent of the cultural gap, levels of education also directly predict economic outcomes.
5. The time frame in which relationships and effects are measured varies. For example, the effects of diversity may be negative in the short term, but positive in the longer term [75].
6. The scale of the relationships and effects vary. For example, organizations, cities, regions or countries [76].
7. The range of diversity may be biased in terms of the samples used (often WEIRD societies [39,41,77]), types of diversity studied, and outcomes of interest; all also shaped by the diversity of researchers and research teams [42].
8. Factors such as discrimination are often ignored in straightforward tests of the relationship between diversity and various outcomes [78].
9. Results may not generalize. For example, findings in one organization and work context with different compositions of diversity along different dimensions (e.g. educational background, identity) may not generalize to another.

We argue that the paradox of diversity emerges as a result of recombinatorial potential on the one hand and coordination challenges on the other. This paradox partially overlaps with the way diversity is often used in public discourses, where it is often characterized by differences in skin colour, ethnic origin, religion, sex, gender, sexual orientation, or ability. Here, we specifically focus on cultural trait diversity, which can but does not necessarily correlate with these other characteristics. For example, Americans with different ancestries may possess similar WEIRD psychology [79]. Cultural trait diversity also partially overlaps with challenging aspects of psychology, norms and institutions, such as racism, prejudice, xenophobia, sexism, other forms of discrimination, power differences, and social and economic inequalities. Here, we specifically focus on coordination challenges, which influence and are influenced by these problematic features of the world. Our goal is to review the overall patterns in the literature and make sense of these in light of cultural evolvability, discussing other aspects of diversity where relevant. We begin with reviews of the effect of different types of diversity in different settings.

Within countries, diversity is often approximated by birthplace diversity, professional diversity, ethnic diversity or linguistic diversity. Research looking at the relationship between diversity and economic growth suggests a positive effect of birthplace diversity, but negative effect of ethnic and linguistic diversity [80,81]. Within cities, greater professional diversity predicts greater productivity [82], but greater ethnolinguistic diversity is associated with greater social tension and conflict [83].

Looking at a specific case, Moser & San [84] show that the 1924 Quota Act, preventing Eastern and Southern Europeans from coming to the USA, is associated with a decline in US scientific innovation. This is consistent with other analyses of the effect of European migration to the USA in the age of mass migration (1850–1920); counties with more immigrants have higher income, less poverty, lower unemployment and greater educational attainment. On the other hand, at least in the short term, culturally diverse communities are less trusting [85,86].

Within organizations, one review showed more innovative teams are comprised of people with more diversity in educational background (measured as the subject of major or degree) and occupational background (e.g. finance, marketing, business development), but more diversity in race and sex had a weak negative relationship [87]. Another review revealed that more innovative firms are comprised of people with more diversity in education and gender, with no effect of ethnic diversity [88]. A meta-analysis revealed that deep-level diversity (such as personality, values and attitudes) was positively related to team creativity and innovation, but surface-level diversity (such as nationality, race and ethnicity) was negatively related or unrelated [89].

Looking at acculturation at the firm level, employees who show indications of acculturating to the organizational culture, such as through language, are more likely to be promoted and less likely to involuntarily exit [90]. Mergers and acquisitions often fail, and the failure is often attributed to the poor cultural fit of the merged organizations [75]. In an experimental test of this hypothesis, Weber & Camerer [91] created lab ‘firms’ with separate organizational cultures created through idiosyncratic language which then ‘merged’. Differences in language reduced performance.

Within teams, measurements of the effect of existing diversity on team performance often reveal mixed effects [92,93]. In an experimental test looking at group size, composite diversity (a composite measure of demographic, professional, psychological and relational variables) and team performance (measured by score on a geo-political forecasting challenge), Pescetelli *et al*. [94] show that more diverse teams increased team performance in larger groups, but harmed performance in smaller groups.

Finally, within science, high-impact papers and technologies are often the result of conventional and atypical combinations of literature and patents, respectively; that is, grounded in one discipline or domain but borrowing solutions from another [95,96]. But of course, by looking at published papers and patented technologies, these data do not capture the many failed collaborations caused by the challenges of disciplinary differences.

Together, these reviews and examples paint a mixed picture of the effect of diversity on performance, innovation and economic growth. Cultural evolvability can help make sense of these findings and perhaps guide future research on these additional challenges. Here, we explore some of these insights and future directions.

### (a) Cultural evolvability means tolerance for diversity

Cultural evolvability means tolerance for diversity, because currently less adaptive traits may be more adaptive when the environment changes. Across societies, a useful measure of this tolerance is tightness and looseness: the degree to which norms are followed and enforced [97,98]. In tight societies, such as many Asian countries, norm violations are met with harsh punishments. Such measures make sense when costs of deviation are higher, such as when there are threats to material security [99–102]. Deviations will tend to be less adaptive than the majority strategy. In such societies, not following a successful ‘Tiger Mother’ [103] type strategy—working hard to secure scarce educational opportunities and subsequent employment opportunities—has a much larger cost. Tighter societies are associated with incremental innovation and loose societies with radical innovation [104,105]. If you conform to the majority, deviations are likely to be smaller. If you conform less, deviations are likely to be larger. Thus cultural evolvability means under-optimization and inequality.

### (b) Cultural evolvability means under-optimization and inequality

Cultural evolvability necessarily means inequality in outcomes, because not all will have the optimal strategy for the current environment. Organizations, for example, face a trade-off between strategies that favour efficiency and strategies that favour flexibility. Early attempts to model this trade-off include Tushman & Romanelli [106] and Lant & Mezias [107]. Organizations increase efficiency through consistent, strong cultures that restrict change. Strong cultures enhance firm performance by improving coordination, sharing similar goals and maximizing the effort of employees. This strategy performs well in stable markets, but poorly during times of change. An analysis of a range of organizations across 18 industries reveals that strong cultures are outcompeted by flexible, more diverse cultures during volatile times [108].

Thus under-optimizing and allowing for flexibility increases an organization's evolvability, allowing them to better adapt to changing market conditions in the longer term. Of course, not all organizations can bear the cost of under-optimizing in the short term—high risk, high value approaches may be better suited to larger organizations or larger countries.

Approaches that take advantage of cultural evolvability include high-risk, high reward skunkworks (i.e. restricting the approach to a part of the company), an ecosystem of different firms trying different strategies (e.g. Silicon Valley), or countries composed of different states or regions trying different approaches (e.g. what US Supreme Court Justice Louis Brandeis described as ‘laboratories of democracy’). Similarly, in programming, and more specifically shared multi-agent reinforcement learning, diversity has shown to increase problem-solving performance through exploration and individualized behaviours [109]. Cultural evolvability means many approaches will be suboptimal or even fail, but the successful approaches can be spread and benefit the group as a whole. Indeed one of the benefits of access to multiple cultures in pluralistic, multicultural societies is the ability to forge new approaches by learning, borrowing, and recombining traits associated with success. If under-optimizing increases innovation, then why are not all countries and companies using this approach to innovation?

### (c) Cultural evolvability helps explain levels of entrepreneurship

Cultural evolvability requires doing something different. Similarly, innovation and entrepreneurship mean deviating from the status quo. People and organizations vary in their willingness to take an entrepreneurial risk. Most new businesses fail and the willingness to take a risk depends on personal and population-level costs and benefits.

First is the personal cost of deviation; many deviations will result in lower payoffs than following the majority trait. If it were obvious how to do better, most of the population would already use the better strategy. Tolerating diversity in traits, thus, means tolerating failure. Reducing the cost of failure increases entrepreneurship as shown for bankruptcy laws and social safety nets, all of which increase entrepreneurship and innovation [110–113]. One of the best predictors of being an inventor in the USA is having rich parents—a child with parents in the top 1% income distribution is 10 times more likely to be an inventor than a child born below the median, controlling for measures of ability [114]. Explanations for this finding include exposure to innovation, access to well-connected individuals, but also the financial resources and safety net, which wealth provides. When failure causes you to fall, you must not fall too far.

Second is the potential population-level benefit of deviation. In a large economy with a large customer base comes large rewards for large innovations—the few winners can win bigger. Amazon can make more money in the USA than in Australia. This logic is captured by a model of overconfidence by Johnson & Fowler [115]. Overconfidence that leads to competing, a proxy for entrepreneurship, is adaptive when the ratio of the benefit of success to the cost of failure is sufficiently high. It is fine to keep losing as long as your occasional wins are greater than your losses. But we can apply this logic beyond the model to a population level, even when individual benefits do not outweigh individual costs at an individual level.

Third is who pays the cost and who benefits from the innovation at a population level. This is in part a function of the scale of cooperation [20]. That is, even if at an individual level the benefits of entrepreneurship do not outweigh costs, they may do so at a population level if the innovation leaves everyone better off. Larger countries and companies can tolerate more diversity and deviation than can smaller countries and companies who may be better off copying and sticking to a successful script.

Silicon Valley offers an example. For every Apple and Amazon, there are thousands of start-ups that have failed—most start-ups fail [116] and the overwhelming majority never receive funding [117]—‘unicorns’ are called unicorns for a reason. Many of those entrepreneurs would have had higher lifetime earnings by taking a salaried job. But the few successes pay for the failures in an investor's portfolio and at a population level. And for this reason, the population may develop a culture of individualism, non-conformity [118–120], and overconfidence [121,122]. Indeed, risk-taking and overconfidence can evolve through success-biased transmission as people see the successful but not the unsuccessful [118,123].

To be an entrepreneur requires a willingness to deviate from the majority and a belief that you are not only better than other potential entrepreneurs (overplacement), but confidence in that belief (overprecision) [124,125]. Without overconfidence in overconfidence, one may end up a ‘wantrapreneur’, holding the belief that one would succeed as an entrepreneur but not being sufficiently confident in the belief to take the risk. Thus, looseness as a cultural package can encourage diversity and that diversity creates more radical innovation, but also more inequality in outcomes. The redistribution of the payoffs that emerge from these different strategies, and thus reducing inequalities in outcomes, is a key factor to resolve the paradox of diversity.

In tolerating failures, societies face the trade-off between the costs of bankruptcies and social safety nets, and collective benefits from the risks entrepreneurs take. For this to work, directly or indirectly, the rewards from innovation must be redistributed and greater than the cost borne by the society for the many failures. This redistribution may be direct, for example, through taxes, or indirect, for example, by increasing efficiency or improving payoffs for other companies and the people who benefit from them. Ironically, however, although tight societies discourage diversity, that intolerance of diversity can create polarization and a kind of ‘cultural speciation’.

### (d) Cultural evolvability can prevent polarization and cultural speciation

Cultural evolvability interacts with the strength of norm enforcement. Tighter societies are associated with greater intolerance for deviation from social norms. But ironically, as Michaeli & Spiro [126,127] theoretically and empirically demonstrate, harsh punishments for minor deviations can increase extremism and polarization. We argue that this polarization in turn may create new cultural groups with more culturally distant cultural traits; a kind of cultural speciation.

The logic of Michaeli and Spiro's main model is as follows: assume a given society holds a social norm that ascribes a behaviour or expressed belief to be correct in a particular domain: for example, all must attend weekly religious services. In a diverse society, some individuals will have desired behaviours and hold personal beliefs that deviate from such a social norm—for example, some may prefer to attend religious services less frequently or not at all. The strength of enforcement of the social norm incentivises individuals to adjust their behaviour and expressed beliefs to different degrees. Societies may vary in the strength of the sanctions (for example, weak sanctions may include withholding help, strong sanctions may include violence) and in the relationship between the strength of the sanctions and the size of the deviation. Both the magnitude of the social sanctions (e.g. lack of approval or punishment) for deviation and the curvature of the function that defines the relationship between sanctions and deviation play an important role in the evolution of diversity in society.

**Figure 3.**
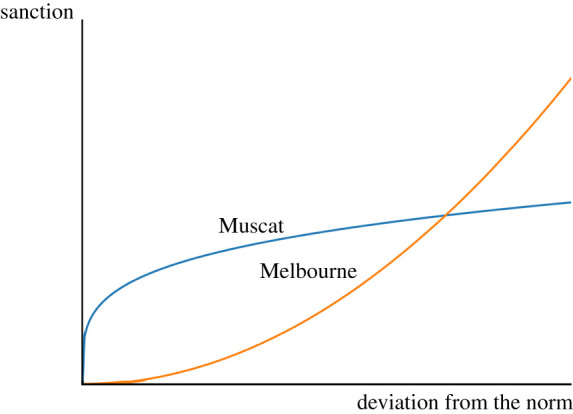
Illustrative example of relationship between deviation from the norm and size of the sanctions based on public goods game experimental data as illustrated in Fig. 1 in Michaeli & Spiro [126]. In cities such as Muscat, small deviations from the norm are punished followed by decreasing marginal sanctions. In cities such as Melbourne the opposite occurs, as they do not punish small deviations, but increasingly punish larger deviations . Thus, sanctioning can be both harsh and concave (Muscat), or harsh and convex (Melbourne). (Online version in colour.)

As an illustration, Muscat, Oman is very strict in sanctioning even small deviations from many social norms including religious observance (based on the World Values Survey). We would expect a small difference in the size of the sanction between a small and a large deviation. By contrast, Melbourne, Australia is more liberal and we may expect small sanctions for small norm violations, but has larger sanctions for large deviations. We stylistically illustrate these contrasts in figure 3.

Michaeli and Spiro's model predicts that large sanctions for even small deviations (e.g. Muscat) will create an all or nothing mentality in which individuals either fully conform, or do not conform at all. A person with weakly held private desired behaviours or non-conforming beliefs will conform. A person with more strongly held private desired behaviours or non-conforming beliefs will not conform since there is little incentive for compromise if even small deviations elicit a similarly large sanction. By contrast, more tolerance for some deviation (e.g. Melbourne) creates a ‘compromise mentality’ in which individuals conform closer to the norm to a greater extent.

The authors test their predictions using data from the World Values Survey [128] using religious practices and religious norms. Societies with stronger religious norms (e.g. measured by the item ‘the only acceptable religion is my religion’) are associated with polarized religious practice. That is, in societies with stronger religious norms, a larger share of the society either fully follows the norm (e.g. praying five times a day) or deviates strongly (e.g. does not pray at all).

The model and results also have implications for debates on freedom of speech, predicting that large sanctions for small deviations may encourage polarization of opinion. Michaeli and Spiro did not model the evolutionary dynamics of the next generation learning from a polarized rather than evenly distributed more moderate range of cultural traits, but intuitively, this may create the conditions for cultural speciation, where some individuals follow one set of norms and others follow a different set of norms.

Thus, looser societies that tolerate a multicultural diversity of opinions and cultural traits may prevent polarization, and help to reduce the coordination costs of cultural trait diversity. A comparably loose approach to norm enforcement and acculturation may also prevent averse dynamics that play out between communities. For example, native Germans are more likely to enforce social norms on ethnic minorities who do not follow local norms [129]. This clustering of culture complicates a straightforward understanding of cultural evolvability.

### (e) Cultural evolvability depends on cultural clustering

Cultural evolvability depends on how traits are distributed. For example, the same diversity (in terms of frequency of cultural traits) can be maximally diffuse such that individuals themselves possess a great diversity of traits [54,57,130]. Or they can be regionally or ethnically diffuse, such that sampling from different ethnicities or regions looks similar—i.e. the cultural distance between ethnicities or regions are small. Or at the other extreme, the same diversity can be highly clustered within groups and regions (consider the smaller regional distances in the USA compared to Europe [42]).

Cultural groups, defined as clustered cultural traits, may be created by many different processes. For example, conformist learning and learning from common sources [131–133], norm enforcement [134,135], symbolic markers of in-group membership [136], collective memories [137,138], and the forces of cultural-group selection. These forces of cultural-group selection [20,139,140] include:

1. Assortative migration: biases in where people with particular traits move—e.g. more individualist people moving to more individualist countries.
2. Demographic growth: some traits spreading due to their effect on the fertility of those who possess them—e.g. norms that encourage fertility, or their correlation with these traits.
3. Differential survival—some traits helping those who possess them to survive better—e.g. norms that encourage caring for ingroup members or norms that lead to success during the intergroup conflict.
4. Prestige-biased group selection—copying the traits of successful groups—e.g. the spread of American culture through Hollywood.

Competition between internally cooperative, culturally distant, cultural groups can create corruption and undermine democratic decision-making [20–22]. For example, favouring one's kin group or tribe can undermine governmental institutions manifesting as nepotism [41,141]. Similarly, when there is agreement on goals, groups can coordinate on picking the best person or political party to implement those shared goals. But a greater difference in goals encourages supporting the person in your cultural group rather than the best person.

There are several convergent lines of evidence that reveal the challenge of clustered diversity. For example, Africa's colonial history left many nations with arbitrarily drawn national borders that do not reflect traditional or ethnic boundaries. Countries containing different ethnic groups or ethnic fractionalization have more civil conflicts [142,143]. Moreover, within both corporations [144] and countries [143,145,146], moderate, clustered diversity is associated with greater conflict. In highly homogenous groups, people tend to agree on fundamental issues, and in highly diverse groups, each group does not have a sufficiently large critical mass to outcompete other groups with differing interests. Between these extremes lies a zone of cultural-group conflict.

Schnell *et al.* [147] model the competition between cooperative groups at different scales, revealing the importance of resource availability to cooperation and competition. People cooperate to access resources that they would not be able to access by themselves or in a smaller group. The optimal payoff is at the group size that maximizes the per person payoff. The model looks at how transitions between scales of cooperation can occur. As resources are accessed through cooperation, the effective carrying capacity increases, allowing the society to access more resources more efficiently with more people and better technology (e.g. a small group of hunter–gatherers would have trouble exploiting an oil field even with the necessary technology and know-how). Transitions are easier when the cooperation-unlocked energy and resources create a carrying capacity that overlaps with the minimum number of people required to unlock a larger, but more difficult to access resource. It is difficult to directly transition from wood-fired steam to solar panels.

The model implies that the cost of clustered diversity may be low when resources per person are plentiful or where there is alignment between the incentives of different cultural groups. But the same level of clustered diversity is a potential source of conflict under limited resource availability or even the perception of limited resources. As a stylized example, consider a competitor (or competing group) opening a pizza shop. When resources are plentiful, the market is large, the economy is growing, this can be predictive of your success. It is a signal that the pizza business is booming and you could open a pizza shop and do well by copying the cultural traits associated with a successful pizza business. This incentivises productive competition: working harder to have the best pizza in town. With a large enough market, you could expand into a pizza franchise chain.

By contrast, when resources are limited, the market is small, the economy is zero-sum, someone else's success is predictive of your relative loss. They have taken a piece of the pizza market that you would struggle to get back. This incentivises destructive competition: competing by harming others (e.g. negative reviews for competitors). When the market is large, concerns about tax incentives (inequality concerns) or some groups hiring only ingroup members (intergroup competition), remain mumblings and grumblings. As long as there is a sufficient market for you to also start a successful business. But when this is not the case, those mumblings and grumblings can break out into something more: destructive competition [148].

The ‘Joy of Destruction’ economic game has been used to measure the tendency to engage in destructive competition. In the Joy of Destruction game, two participants are given an endowment. One participant is offered the opportunity to destroy another participant's endowment at some efficiency. For example, the destroyer might pay $2 to take $4 from the other participant [149,150]. There is no in-game incentive to engage in destruction and thus any destruction is a function of out-of-game factors. Levels of destruction are higher in places where resources are more limited. For example, rates of destruction in the Joy of Destruction game are higher in Namibia than Ukraine [149] and higher in low rainfall regions within Namibia than in high rainfall regions [151]. Prediger *et al*. [151] use rainfall as an instrumental variable to causally identify the effect of resource availability on rates of destruction.

In the light of cultural evolvability and these lines of evidence, let us consider the research, methodological tools, and policies that may help resolve the paradox of diversity.

## 4. Resolving the paradox of diversity

Cultural evolvability offers a framework for thinking about the trade-off between recombination and coordination: the paradox of diversity. It also offers paths towards resolution—reaping the benefits of diversity by reducing costs.

Diversity has been central to the success of all complex life on earth. Diversity provides the new traits needed to make life evolvable. Until around 1.2 billion years ago the source of that diversity was mutation—genetic innovation through serendipity and incremental improvement alone. Single cells reproducing by simple replication. The evolution of sexual reproduction unlocked the recombinatorial power of diversity, increasing evolvability and the speed of evolution [152]. Sexually reproducing organisms recombine diverse genetic material to empower genetic evolution. Today, diverse societies recombine diverse cultural traits to empower cultural evolution. For example, large, novel leaps in innovations can emerge through intellectual arbitrage [153]—taking a perspective or solution from one place or discipline and applying it to another. But there are many barriers to cultural traits meeting and recombining.

### (a) Reaping diversity's benefits by reducing costs

We live in an increasingly interconnected and multicultural world [154]. Migration has been a constant feature of the human story [155], but since the late nineteenth century's Age of Mass Migration [156], more people from more culturally distant societies increasingly live side by side. And at a global level, their culturally distant countries of origin are forced to coordinate on global issues as never before.

On a local scale, organizations are now forced to navigate the benefits and challenges of diversity. For example, corporate cultural differences between firms may be a cause of the large rate of failure in business mergers and acquisitions [75,91]. Recent analyses reveal just how much human potential is lost through unequal access to information and adaptive cultural traits [114,157]. The goal of any society or organization should be to reap the benefits of diversity and minimize the costs, thereby maximizing human potential. Drawing on insights from cultural evolution, the collective brain and cultural evolvability, we discuss challenges and insights.

#### (i) Measuring diversity

As a first step to resolving the paradox of diversity, we need robust scientific methods to measure cultural trait diversity and its effect. As we discussed, the definitions and measurement of cultural trait diversity vary between papers and between fields. Muthukrishna *et al.* [42] argue that a cultural fixation index (CFst) offers a robust, theoretically derived measure of cultural distance grounded in cultural evolution.

Just as a genetic fixation index (Fst) is theoretically meaningful within population genetics, because it measures how genotype frequencies between subpopulations differ from expectations if there were random mating over the entire population, a cultural fixation index (CFst) measures how cultural trait frequencies between subpopulations differ from expectations if there were broad social learning across the entire population and no selection, migration, and between-group differentiation between subpopulations. As such, CFst allows us to measure cultural distance in a fine-grained and direct manner.

Using CFst, we can thus identify the degree of diversity between any groups, identifying the degree to which they represent different cultural groups. For example, Handley & Mathew [158] show that larger cultural distance, as measured by CFst, predicts lower intergroup cooperation in four pastoralist ethnic groups in Kenya. This is consistent with theoretical work on the evolution of ethnic markers to distinguish group identities to decide who to cooperate with [136,159,160]. Muthukrishna *et al*. [42] focus on cultural distance between regions and countries. White *et al*. [79] focus on cultural distance between religions. Handley and Mathew focus on cultural distance between pastoralist ethnic groups. New, larger datasets, such as those derived from social media [161], will allow for cultural distance to be studied on a much larger and finer scale. These approaches may be able to re-examine past findings on the U-shaped relationship between cultural clustering and trust [145] and cultural clustering and conflict [143,146].

However, as discussed, cultural distance alone is not necessarily a problem. But under resource scarcity or even perceived resource scarcity, it can be. Thus, managing resource availability and perceptions of resource availability are critical to managing the paradox of diversity's pernicious effects.

#### (ii) Resource competition and zero-sum perceptions

Resource competition shapes the effect of diversity. When the perception and reality of competition between cultural groups are positive-sum or resources are perceived to be plentiful, clustered diversity can be a source of strength as separate cultural groups coordinate, productively compete, and cooperate to mutual benefit, unlocking more resources. Clustered diversity may be optimal for avoiding homogeneity through conformity; dividing a problem to solve it [14]. By contrast, when the perception or reality of competition is zero-sum or resources are perceived to be limited, people cooperate at the scale needed to best access those limited resources, creating conflict and destructive competition [147]. This insight has policy implications.

Within organizations, the local context of competition is often under management's control. A case study in the creation of zero-sum competition is Enron and then CEO Jeffrey Skilling's ‘rank and yank’ policy [162]. Employees were ranked on relative performance scoring and the lowest-ranked lost their job. That is, regardless of absolute performance, if you were relatively worse than other colleagues under a predefined threshold, you would be fired [163]. Such a strategy creates zero-sum competition among employees, reducing the scale of cooperation, the willingness to share recombinable knowledge, and facilitates unethical behaviour for personal benefit [164].

Within countries, a review by Baldassarri & Abascal [165] reveals the importance of economic conditions and economic interdependence between groups. Prosocial attitudes are greater under more favorable economic conditions and greater economic interdependence. Consistent with zero-sum perceptions, a review by Craig *et al*. [166] reveals that the relationship between intergroup contact and intergroup relations between majority whites and minorities is moderated by zero-sum perceptions of demographic growth. That is, the majority is threatened by minorities growing relative to the majority. But the context of competition is sometimes under our control. For example, investment in public infrastructure (e.g. schools, hospitals) that matches levels of immigration can mitigate intergroup hostility.

Finally, at a global level, the rhetoric on climate change policy has evolved from zero-sum framing in terms of limits on growth to a more positive-sum focus on sustainable growth and private and collective benefits, reducing perceived inter-country competition [167].

To summarize, under conditions of plentiful resources, clustered diversity is not necessarily an issue and may be helpful as different groups align incentives, specialize and exploit comparative advantages [10,14,168], increasing cultural evolvability.

#### (iii) Bridging the cultural gap

Another obvious key to resolving the paradox of diversity is finding common ground between cultural groups. There are many strategies to achieve this common ground. A basic requirement is communication across diverse groups and common sources of information.

Language is probably the most obvious dimension that affects communication—native language proficiency increases employment probability and earnings [169]; beyond coordination, earning differences may result from discrimination based on accents [170]. Investment in language programmes can help close this earnings gap [171,172]. But other cultural traits can also impede communication and coordination and increase discrimination, reinforcing intergroup inequality. An optimal strategy would involve identifying which cultural traits and cultural dimensions policies may target to ensure the best outcomes for both migrants and locals. For example, individuals and groups can act as translators and bridges—dual language speakers, individuals trained in multiple disciplines, or communities who have a cultural overlap between two other communities. The development of real-time translation software shows that technology offers further potential to help bridge the cultural gap.

More generally, formal education serves as a means by which a cultural package is efficiently transmitted between generations. Thus, unequal access to education, between and within societies, creates a cultural gap that is difficult to close without increasing access to shared education sources. Indeed, the cultural distance between those with higher education in different societies is likely to be smaller than the cultural distance between those with lower education. Moreover, given the importance of education as a means of cultural transmission, the cultural distance between societies may also reflect educational and economic differences that equally affect the cultural gap.

The specifics of cultural traits matter and may be incompatible. Such traits may range from which side of the road one drives on, to whether your marriage practices include bride prices, dowries, or no material transfer from either side to power distances and equality between sexes, to different world views created by different amounts and types of education. These are important, but difficult challenges, especially since cultural traits are not independent, but connected to one another in ‘cultural complexes’ of mutually interdependent cultural traits, analogous to gene complexes [173].

Migration is a boon to economic development [174], but these overall results differ by cultural, economic and educational distance. As one example, first-generation European migrants to the UK make a greater fiscal contribution relative to their cost to the social welfare system than do non-European migrants (who are similar to locals in their contribution to cost ratio) [175]. In an analysis including the UK, France and Germany, and both first- and second-generation migrants, Algan *et al*. [176] show similar differences between culturally close and culturally distant ethnic groups. Between generations, the education and language gap closes in most cases. The employment gap decreases, but does not close.

There are many caveats to interpreting these studies. In the second generation, it is more difficult to identify ethnicity and immigration status creating possible sampling biases. But even in the first generation, discrimination and other barriers faced by more culturally distant migrants likely contribute to these differences. However, a straightforward application of this empirical literature to policy would preference more culturally close migrants. By contrast, from a cultural evolvability perspective, it is more culturally *distant* migrants who offer more radically different cultural traits for recombination. Thus, from this perspective policies that target the challenges faced by new migrants, particularly those from more culturally distant places of origin, and particularly those that target cultural traits that harm communication and coordination, are likely to reap benefits. The key in this case and others is reducing communication and coordination costs to unlock the benefits of diversity for all members of society. There are many ways for this to be achieved.

Within organizations, Cremer *et al*. [177] show that managers can serve as translators, supporting between-unit coordination where units lack a common technical language. Groups can also negotiate a middle ground through increased perspective-taking, which is associated with higher team creativity [178].

Within countries, where diversity is less clustered, people may be forced to find common ways of communicating. For example, people from countries with a long history of migration use more universally recognizable facial emotional expressions [179,180]—for example, Americans are known for their broad smiles and unambiguous displays of emotion. In contrast, in a more homogenous country like Japan, other Japanese may understand emotion based on context without the need for explicit expression. But if your neighbour comes from a very different place and does not speak the same language, you need to be explicit in your emotional expression.

Research on contact theory reveals that a collaborative rather than adversarial contact—consistent with positive rather than zero-sum conditions—decreases intergroup differences and hostility [181]. But the dimensions of cultural difference matter and results on contact theory do not necessarily generalize. For example, in a field experiment, Mousa [182] increased contact between Christians and Muslims in football teams, raising tolerance, but not overall social cohesion. Because the specifics of cultural trait diversity matter, directly measuring cultural distance, trait and dimension differences, and considering time scale and cohort are important aspects of resolving the paradox of diversity. Time is particularly important; many studies show fleeting or ambiguous effects of contact [183]. Groups need time to find ways to communicate and coordinate [184]. Moreover, small interventions over short time periods (such as short-term bias training; for review see [185]) are unlikely to remove the underlying causes of barriers to communication and coordination nor the different levels of discrimination that different groups face.

Finally, an important future area of research is how new forms of communication, such as the Internet and social media, and new forms of meeting and networking, such as social media and dating apps affect the paradox of diversity.

## 5. Conclusion

Humans are a deeply cooperative species. Our greatest achievements and our worst atrocities are both cooperative acts. The scale of our cooperation has increased over time, but still varies considerably between groups [20,48]. Through large-scale cooperation, we share ideas and allow our societies' collective brains to innovate solutions to problems we all face. That innovation is empowered by diversity, but that diversity also by definition divides groups into smaller cooperative groups with lower levels of trust and the ability to communicate, coordinate and work together for mutual benefit. The challenge is greater in a world in which more culturally distant people live side by side and in which more culturally distant societies must coordinate on global challenges. But while the challenge is greater, so too are the potential gains.

Cultural evolvability offers a framework for understanding the importance and impact of diversity and how to reap its benefits and reduce its costs. By resolving the paradox of diversity, we bring more perspectives to bear on our common problems, encourage recombination of the best solutions from different societies and disciplines, and unlock human potential by creating conditions conducive to ever larger scales of cooperation and ever greater levels of innovation.

## Endnotes

1 Analogous to the concept of effective population size in population genetics, here a function of the effective number of models for learning.

2 Some insights from machine learning are particularly interesting. For example, getting stuck in local optima is sometimes considered a problem. However, in a sufficiently high-dimensional space there are effectively no local optima, only saddle points with some dimension that allows escape. Given the large dimensionality of biological and cultural systems, there may be no true evolutionary stable equilibria.

## Data accessibility

Code is submitted as electronic supplementary material.
